# Supplementary figures and images for: A Novel Strategy for Constructing an Integrated Linkage Map in an F1 Hybrid Population of Populus deltoides and Populus simonii
Source: Genes (Basel). 2022 Sep 26;13(10):1731. doi: 10.3390/genes13101731 (PMC9601732; doi:10.3390/genes13101731)

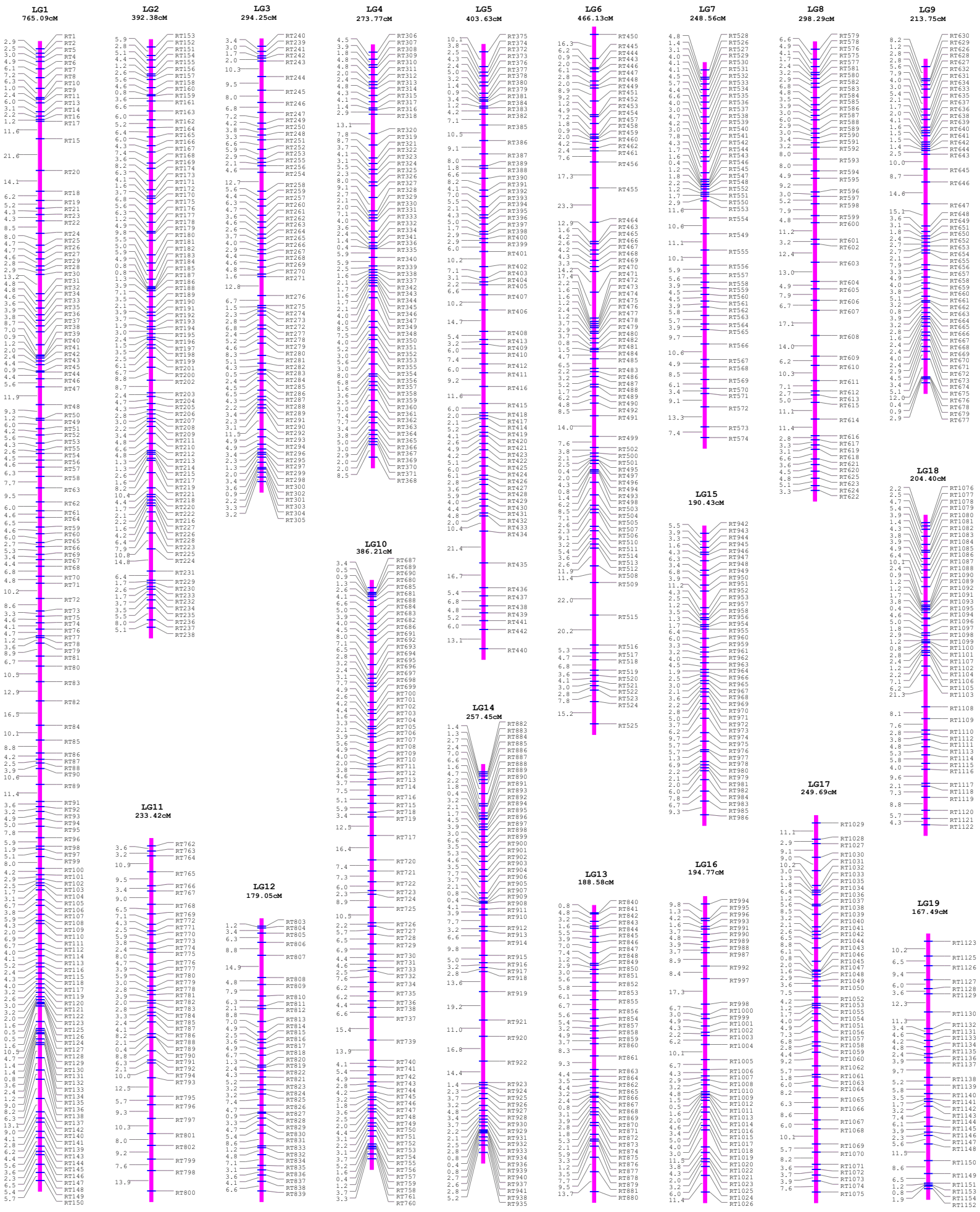

Supplement: Supplementary file 1 [file genes-13-01731-s001.zip › Figure S1.pdf]

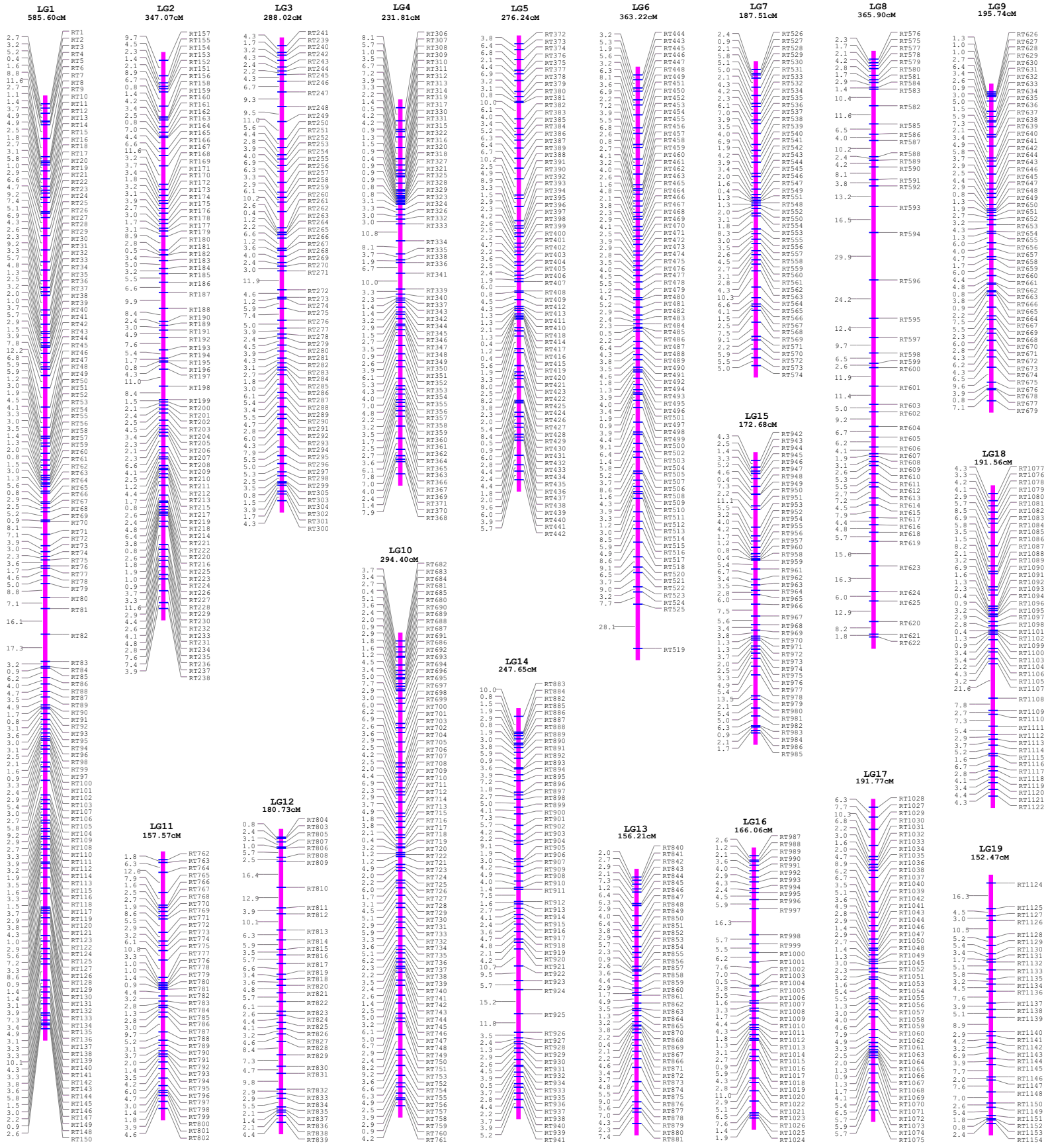

Supplement: Supplementary file 1 [file genes-13-01731-s001.zip › Figure S2.pdf]
